# Pipes and Potions: Testing the Efficacy of European Folk Preparation Methods for Anticholinergic Solanaceae Plants

**DOI:** 10.3390/plants11010126

**Published:** 2022-01-04

**Authors:** Karsten Fatur, Matjaž Ravnikar, Vitjan Fras, Samo Kreft

**Affiliations:** Faculty of Pharmacy, University of Ljubljana, 1000 Ljubljana, Slovenia; Matjaz.Ravnikar@ffa.uni-lj.si (M.R.); vitjan.fras@gmail.com (V.F.); samo.kreft@ffa.uni-lj.si (S.K.)

**Keywords:** Solanaceae, anticholinergic alkaloids, ethnobotany, traditional knowledge, ethnopharmacology

## Abstract

The present article sought to evaluate the efficiency of various folk preparation methods commonly used in Europe for employing anticholinergic Solanaceae plants. The study aimed to uncover which folk methods were effective for the extraction of the anticholinergic tropane alkaloids of these plants, atropine and scopolamine. The folk extractions that were tested sought to simulate the preparation of teas, cold-water infusions, unguents, tinctures, fortified wines, and smoking. All preparation types and a control were then put through an extraction process to see what amount of the alkaloids had been maintained. These extractions were then analysed using high-performance liquid chromatography (HPLC). Cold- and hot-water preparations, tinctures, and fortified wines all proved to be effective means of extracting atropine and scopolamine from plant material under conditions seen in folk usage. Smoking and the oil-based unguent, however, yielded no alkaloids, suggesting a lack of efficiency for these preparations, a problem with our methodology, or possible chemical changes and losses associated with the preparation procedure.

## 1. Introduction

Folk preparations for medicinal plants take on a range of different forms depending on their intended usage as well as the preferences and traditions of those making them. A single plant may, in fact, produce many medicines once it is considered how many different forms of preparation exist and how many parts of a given plant may be used. This becomes an increasingly important consideration when speaking of medicinal plants that are toxic in concentrations that could reasonably be consumed by an individual, either accidentally or purposefully.

**Figure 1:**
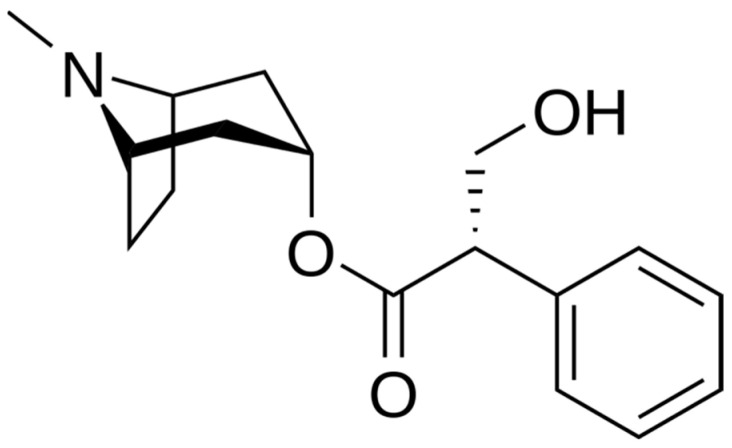
Chemical structure of hyoscyamine. Public domain.

**Figure 2:**
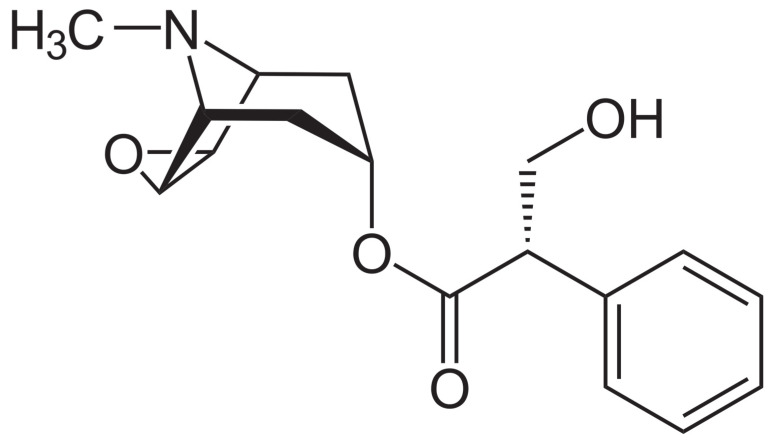
Chemical structure of scopolamine. Public domain.

One such group of plants are those in the Solanaceae containing anticholinergic tropane alkaloids; sometimes referred to as the nightshades and represented by the genera *Atropa*, *Datura*, *Hyoscyamus*, *Mandragora,* and *Scopolia*, these plants have played an important role in the herbal and medicinal histories of Europe. These plants contain hyoscyamine ([C_17_H_23_NO_3_, Figure 1] better known as atropine when in its racemic form) and scopolamine (C_17_H_21_NO_4_, Figure 2), both of which are powerful muscarine antagonists that prevent the binding of acetylcholine, thus causing a range of effects related to heart rate, perspiration, respiration, smooth muscle contraction, and central nervous system function [1,2,3,4,5]. As a result, these plants have been extensively used medicinally as well as recreationally throughout European history, as well as having been employed for hunting, as poisons, and for a range of other important cultural purposes [6]. Medicinally, these plants were primarily used for their anaesthetic and analgesic effects, though this use came to be largely discontinued in favour of using them for treating asthma and other respiratory ailments; in addition, they also found use for many other health concerns, ranging from skin conditions to demonic possession [6].

Though some modern medicinal usage continues for these plants, they are now primarily used as recreational substances, often with dangerous outcomes. This recreational usage is due to the ability of hyoscyamine/atropine and scopolamine to cross the blood-brain barrier and to produce vivid, life-like hallucinations and altered mental states, though usually with dark undertones and serious consequences; as a result of their unique effects, these substances are classified as deliriogens [2,5,7,8].

*Scopolia carniolica* Jacq., the plant used in this present research, is used extensively in industry today as a source of tropane alkaloids for industrial extraction [9]. In addition to this, however, it is also known to have been used as an intoxicant in Europe, a traditional practice that continued at least into the last century in the Baltic nations and that continues in Slovenia and other parts of Europe to a certain extent even now [10,11,12]. This plant is also known to have a history of medicinal use, having been employed in Slovenia for the treatment of skin problems and in the Baltic nations as an anti-paralytic and abortifacient [10,13].

This difference between recreational and therapeutic effects is likely linked not only directly to dosing, but also to preparation methods and the effectiveness with which they make the alkaloids of the plants available. We set out to examine the alkaloid content of samples prepared as hot- and cold-water infusions, unguents, fortified wines, tinctures and for smoking, which are the traditional methods of preparation seen in Europe over the past centuries.

## 2. Materials and Methods

### 2.1. Sample Collection

Samples of *Scopolia carniolica* (English: henbane bell, Slovene: kranjska bunika) were selected for use in the present research due to their proven content of atropine and scopolamine in a previous project in our research group [14]; a number of other species from the aforementioned genera in the Solanaceae, however, could also have been used. The samples were collected while the plants were in flower, between 1–9 April 2019 and 24 March–4 April 2020. Voucher specimens (KFSC2019 1-4) were made for the collections and deposited into the herbarium collections of the department of pharmaceutical biology at the Faculty of Pharmacy, University of Ljubljana. The Latin name for the plant was verified using theplantlist.org and is accurate as of 31 December 2021.

### 2.2. Sample Preparation

Samples were dried at room temperature and divided based on organ type (flowers, leaves, and roots). The dried samples were ground and used in our previously mentioned study [14]. After the completion of this work, the remaining samples were mixed together by organ to ensure that there would be a large enough quantity of homogenous sample for the different tests to be carried out in the present research. As such, the samples used in this paper represent a compilation of the samples obtained from many different plants, having been collected from three sites (46°02′21.7″ N 14°30′50.1″ E, 46°04′26.1″ N 15°27′03.5″ E and 45°53′03.8″ N 14°22′02.9″ E). This left our group with two samples: *S. carniolica* root powder and *S. carniolica* leaf powder. Root powder was used in for all extractions except for the smoking trials, where the leaf powder was used after the root powder proved resistant to burning. Three independent replicate extractions of each type were prepared.

### 2.3. Folk Extractions

#### 2.3.1. “Tea” (Hot- and Cold-Water Extractions)

Six samples were prepared to simulate tea and water extracts; hot and cold water were each used, with steeping times of 10, 20, and 30 min for each. 0.25 g of powdered root was steeped in 50 mL of water for each sample. Hot water was brought to a boil and then poured onto the samples, which were not on heating elements, trying to most accurately simulate the process by which teas are made.

These samples were filtered after their allotted time span in order to remove the herbal drug. Each water extract was acidified to pH 2 using 1.7 mL of 0.5% HCl and poured into a dividing funnel. 3 × 20 mL of dichloromethane was used to wash the sample and then discarded. This was followed by the addition of 200 μl of 30–33% ammonium hydroxide to bring the pH to 8–10.

The samples then went into phase 2 of the extraction, as described below in Section 2.3.7.

#### 2.3.2. “Unguent” (Coconut Oil Extractions)

Three samples were prepared to simulate the creation of an ointment/unguent for topical application. Each used deodorised coconut oil brought up to 80 °C, which was then allowed to continue cooking on the burner for either 10, 20, or 30 min once the plant material (root powder) was added. Coconut oil was chosen in accordance with information gained from ethnographic interviews from a previous project in our working group [11]. The mixtures were then immediately filtered and left overnight in order to simulate the process of making an unguent. They were kept in a heated chamber at 40 °C to ensure that they would not solidify and make further extraction and analysis impossible. The following day, the plant material was removed by filtration and the ointments were each extracted with 3 × 10 mL of 0.5% HCl; this acid mixture was kept, and the oil was discarded. 1 mL of 30–33% ammonium hydroxide was then used to bring the pH to 8–10.

The samples then went into phase 2 of the extraction, as described below in Section 2.3.7.

Using the same method, we also attempted an unguent that had been basified using sodium carbonate, though only with a cooking time of 30 min. This extraction was done after witnessing the results of the previous oil extractions.

A re-extraction of the plant material from the non-basified unguent was also carried out using the methods outlined below in Section 2.3.6. for the control samples.

#### 2.3.3. “Fortified Wine” (Wine Extraction)

Three samples were prepared to simulate the cooking of wine fortified with anticholinergic plants in accordance with historical records and ethnographic interviews from previous work in our research group [11]. 50 mL samples of 11.5% Refosco red wine (Vinakoper, Slovenia) were brought to 80 °C before having 0.25 g of root powder added and then being cooked for 10, 20, or 30 min. After removal of the plant material by filtration, the fortified wines were evaporated to 30–60% of the original volume to remove ethanol, and then 0.5% HCl was added until the pH reached 3. They were then washed with 3 × 20 mL of dichloromethane, which was then discarded. The mixtures were then basified using 30–33% ammonium hydroxide until they each reached a pH of 8–10.

The samples then went into phase 2 of the extraction, as described below in Section 2.3.7.

#### 2.3.4. “Tincture” (Alcohol Extraction)

50 mL of 70% ethanol was mixed with 0.25 g of dried root and allowed to sit over a period of 3 days in a beaker covered with parafilm. The mixture was swirled around each day briefly with the overall effect being to simulate the creation of an alcoholic tincture. At the 3-day mark, the mixture was filtered into a round-bottom flask and then evaporated in a rotary evaporator at 175 mbar using a 40 °C water bath. 25 mL of 0.5% HCl was then used to resuspend the dry extract in the flask and to transfer it into a separating funnel. 3 × 20 mL of dichloromethane was then also used to wash the flask into the funnel, with each wash then being discarded. 1 mL of 30–33% ammonium hydroxide was then used to bring the pH to 8–10.

The samples then went into phase 2 of the extraction, as described below in Section 2.3.7.

#### 2.3.5. “Smoking” (Smoke Extraction)

**Figure 3:**
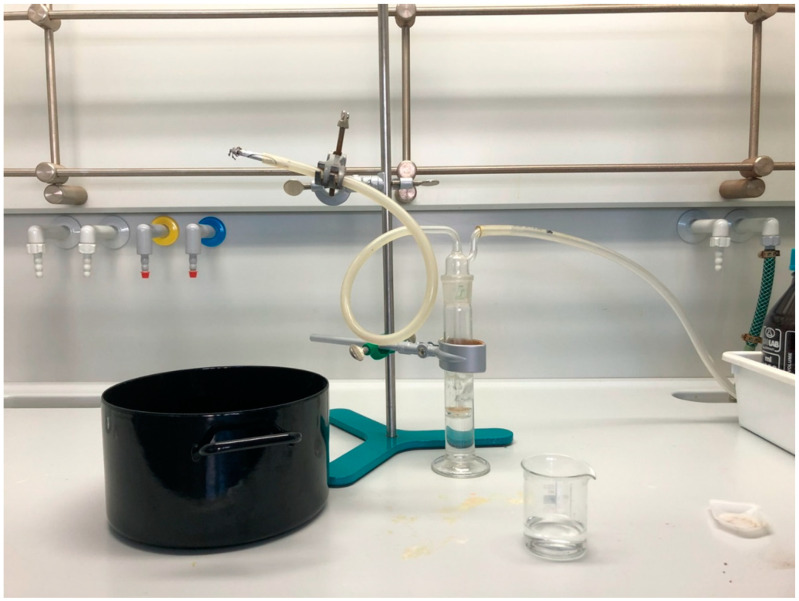
Cigarette smoking simulation setup.

**Figure 4:**
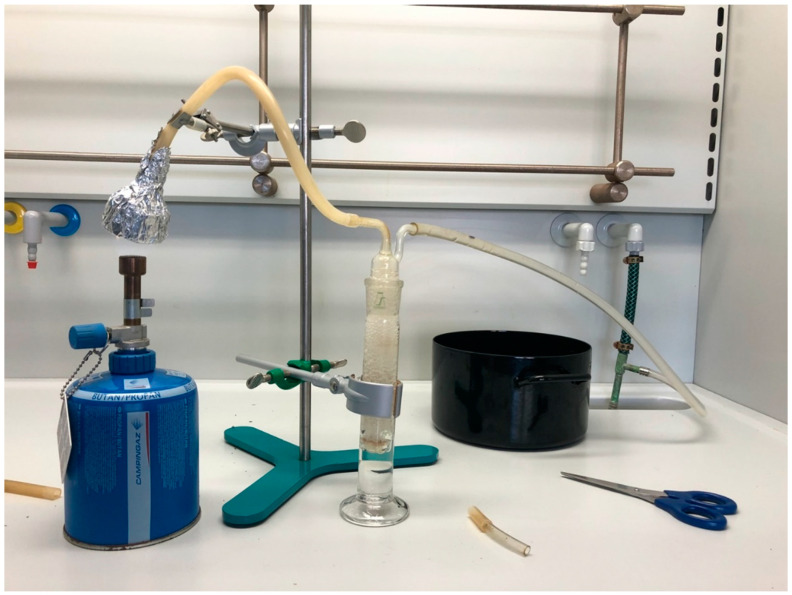
Pipe smoking simulation setup.

Different setups were used to extract alkaloids from smoke. We initially used the root powder so that results would be comparable to the other extractions; however, it proved resistant to burning. We then switched to the powdered leaves. Our initial setup (Figure 3) involved simulating the use of a hand-rolled cigarette. The burn of the cigarettes proved difficult to control and many parts fell off unburnt. As such, we switched to a setup simulating a pipe (Figure 4) that had a constant flame applied in order to create more standard measurements and ensure complete combustion of the plant material. The smoke was then pulled through 50 mL of 0.15% HCl with 20 mL of dichloromethane; since the two substances do not mix, the dichloromethane was introduced to raise the volume, thus allowing the HCl to be on the active part of the apparatus. This dichloromethane was treated as a first wash and was followed by 2 × 20 mL dichloromethane washes, each being discarded after application. 30–33% ammonium hydroxide was then used to bring the pH to 8–10.

The samples then went into phase 2 of the extraction, as described below in Section 2.3.7.

#### 2.3.6. Control Extraction

The control samples for both root and leaf powder were extracted using a modified procedure based on our previous project [14], which followed a process modified from previously published research [15].

0.25 g of powder was washed into a 50 mL beaker with 15 mL of 70% methanol. The beaker was put into an ultrasonic bath without additional heat for 30 min with a parafilm cover with holes poked into it. The mixture was then left at room temperature for 48 h before again being put into the ultrasound bath for 30 min. It was then filtered into a 50 mL round-bottom flask, with 5 mL of methanol being used to wash the beaker into the flask as well. It was then evaporated in a water bath of 40 °C at 200 mbar with a rotary evaporator. 25 mL of 0.5% HCl was then used to wash the flask into a dividing funnel. 3 × 20 mL of dichloromethane was then also used to wash the flask into the funnel, with each wash then being discarded. 1 mL of 30–33% ammonium hydroxide was then used to bring the pH to 8–10.

The samples then went into phase 2 of the extraction, as described below in Section 2.3.7.

#### 2.3.7. Phase 2 Extraction

Extraction continued to follow the path of our group’s previous research, modified from methodology found in the literature [15]. 5 × 20 mL of dichloromethane was sequentially used and collected with each extract. The water phases were then discarded, and 15 g of anhydrous sodium sulfate was then added to the dichloromethane. This was then filtered into a 100 mL point bottom flask with an additional 2 mL of dichloromethane used to rinse the filter. This was evaporated at 700 mbar in a 40 °C water bath with a rotary evaporator. 2 × 2.5 mL of methanol was then used to wash the flask into a test tube from where it was then filtered and prepared for high-performance liquid chromatography (HPLC).

#### 2.3.8. Analysis

Quantification of the compounds and identification via reference standards were carried out through an HPLC system (Shimadzu Prominence) consisting of a system controller (CBM20A), a column oven (CPO-20AC), and a solvent delivery pump with a degasser (DGU20A5) connected to a photodiode array detector (SPD-M20A) monitoring wavelengths 190–800 nm. LabSolution software version 5.71 was used to record the responses of the detectors. Using a Phenomenex Kinetex^®^ C18 column (10 cm × 4.6 mm I.D., 2.7-µm particle size), chromatography was performed at 40 °C with a flow rate of 2 mL min^−1^. We utilised the following gradient method with solvent A (water with 2% acetonitrile and 0.1% trifluoroacetic acid) and solvent B (acetonitrile with 2% water and 0.1% trifluoroacetic acid): 0–1 min 3% B, 1–10 min 20% B, 10–12 min 100% B, and 12–15 min 5% B. Samples and standards were detected at an absorbance of 210 nm. Concentrations of atropine and scopolamine were calculated based on calibration curves made with standard substances. The efficacy of extractions were expressed as % of yield obtained in comparison to the control extraction.

## 3. Results and Discussion

### 3.1. Control

The content of atropine and scopolamine in both of our control mixtures are listed in Table 1. The content of the alkaloids was much higher in the leaves than in the roots, with this difference being especially pronounced in terms of scopolamine concentration. Atropine was the dominant alkaloid in both sample mixtures, but by a much smaller margin in the leaves than in the roots. This is in line with our previous results [14].

### 3.2. “Tea” (Hot- and Cold-Water Extractions)

The “tea” extractions were meant to simulate the use of these plants in water infusions. Though the preparation of these plants as water infusions has been extensively reported in the literature, the details are lacking. As such, we elected to try both cold- and hot-water infusions and chose to examine the effect of steeping time on alkaloid concentration. The results are listed in Table 2.

It can be seen that water and a moderate amount of time are already efficient enough to extract up to 50% of atropine and scopolamine from plant material as a tea. That being said, this may not be beneficial within the context of folk preparations as the water extracts are usually consumed in larger volumes compared to other forms of preparations (e.g., tinctures). Since these alkaloids are also poisonous and can cause a strong state of delirium, preparation would need to find a careful balance between using too much or too little, which would be affected both by preparation and by the content of plant material used to begin with. Indeed, assuming a standard teacup, our present results would predict 1000 µg of atropine and 150 µg of scopolamine to be ingested from a single cup of tea when made with hot water and steeped for 20 min from 2 g of dried plant material. This is well beyond the range of a therapeutic dose and into dangerous consumption [16,17,18].

It is worth noting that water infusions are the least common of the methods here presented to appear in the literature; few historical sources seem to note these plants being employed in this way and some even note that though medicinal, such usage is unwise [19]. One recent study [20] found the use of a plant that may have been *Hyoscyamus niger* as a medicinal tea, but the vast majority of usage as water infusions has centred on intentional intoxication [21]. These are mainly reported in toxicological studies as summarised in the previous reference, which is unsurprising given the dangerously large quantity of alkaloids that one could easily consume in this manner.

The temperature of water had only a minor effect on the extraction of alkaloids. Both hot- and cold-water infusions for both alkaloids displayed a non-linear relationship between concentration and time, with yields being lower at 30 min compared to 20-min extraction time. This is likely attributable to the speed of hydrolysation at differing temperatures [22].

### 3.3. “Unguent” (Coconut Oil Extractions)

Unguents made from plants containing these alkaloids have been well-known since antiquity in Europe for use against a range of ailments, such as ulcers, gout, sores, inflammation, joint and muscle pain, and even male infidelity, to name a but a few; though such ointments were traditionally made from animal fat, coconut oil was mentioned by some who have employed these sorts of unguents in modern times [11,19,23,24,25,26]. Perhaps the most famous stories of these unguents, however, are about their uses in medieval witchcraft as flying ointments. As the stories go, witches would smear these unguents onto their bodies and use them to take flight and reach the demonic sabbaths where they met with the devil; though elements of these stories were extended beyond any semblance of reality in the frenzy of the medieval witch hunts, it is still believed that these ointments were used as a recreational intoxicant among the poor who were unable to afford alcohol and other more costly pleasures [26,27,28,29]. Legends of these sorts of ointments are known around Europe and also have an extensive history within Slovenia and its neighbouring nations [30,31,32].

As with for water extractions, the exact details of preparing these ointments are not clear. We tried 10, 20, and 30-min extractions, but only 30 min yielded some scopolamine in analysis; the results are listed in Table 3. We theorised that the alkaloids would need to be basified in order to be converted into their uncharged form and to be soluble in the oil. Sources in the literature speaking of the medieval witch trials and these ointments often list a range of other ingredients besides just these tropane alkaloid-containing plants; many of these were likely only said to be included in order to make the ointments sound more sinister (for example, human blood, nail clippings, and pubic hairs; baby fat; and the foreskins of newborns) [27,30,33]. However, ash is also mentioned as an ingredient of these ointments and it has been previously suggested that this may have been employed to render the alkaloids more absorbable by the human body, similar to how chewers of coca leaves will sometimes combine them with ash [34]. We believed that if ash were to be used with plants containing these alkaloids, then it may raise the pH enough for the oil to successfully extract the alkaloids. As mentioned in the preceding methods section, we then also tested the extraction in the oil in the presence of sodium carbonate. This, however, still did not allow any atropine or scopolamine to be detected in our ointment.

Additionally, we verified if the alkaloids that did not extract from the plant to the oil remained in the herbal material; our re-extraction of plant material still yielded atropine and scopolamine. Based on our controls, we know that both should have been present to some degree in either the unguent we prepared or in the plant material. As a result of these findings, we hypothesised that perhaps the alkaloids were in fact so strongly stuck in the oil phase that we were unable to extract them and they were thus lost during the experiment when the oil phase was discarded after attempted extraction with dichloromethane. Since scopolamine is many times stronger than atropine in its psychoactive activity and since these ointments were known to cause very intense hallucinations, our lack of results may itself point to the effectiveness of this method [34,35,36,37]. Further research will be needed to better understand the chemical processes at work when creating oil-based unguents with anticholinergic tropane alkaloids.

### 3.4. “Fortified Wine” (Wine Extraction)

The fortifying of wine with Solanaceae plants containing tropane alkaloids has an extensive history in Europe for medicinal, ceremonial, and recreational purposes; extending back as far as ancient Greece, this tradition is still seen to a lesser extent today [11,19,24,38,39,40,41,42,43,44,45,46,47,48].

Once again, these records are non-specific and thus we attempted three different cooking durations. Red wine was used in accordance with information on modern use gained from previous research in our working group [11]. The wine contained its highest concentration of atropine and scopolamine in the sample cooked for 30 min, with lesser amounts found with shorter extraction times; the results are listed in Table 4.

If the wine was meant to be used recreationally, however, it seems likely that it would have been desirable to have scopolamine present, since it is the much more psychoactive alkaloid [35]. The cooking times to include scopolamine, however, also include large amounts of atropine, and so it would be important that individuals consuming these beverages were careful not to overindulge. Users dying from these kinds of wine are not featured in the historical literature, but the propensity in humans to overconsume when intoxicated is widely known. As such, the question remains as to how dosing was measured in recreational settings. In medicine, it is simple to give the patient a certain amount, but a large party is another matter entirely. Since such wines have long moved from the focus of large banquets to more niche consumption, we may never know how this was handled.

It is worth noting that the wine extractions generally yielded more alkaloids than those done in water, especially as time progressed. This, however, may be partly due to the cooking method itself and not only due to the difference in the solvent. The wine extractions were carried out on heat for the entire time, while the cold-water extraction obviously used no heat and the hot-water extraction used heated water added to the plant material and allowed to steep after being removed from the heating element. As such, the difference here is not only wine vs. water, but also that of cooking vs. steeping. Though this makes the results more difficult to directly compare, both were carried out in accordance with historical and ethnographic data to compare actual conditions in which these beverages would be made rather than more hypothetical ones.

### 3.5. “Tincture” (Alcohol Extraction)

Tinctures made from plants containing these alkaloids are widely known to have medicinal applications, both for internal and external usage; though mostly historical, modern usage does continue in some parts of Europe [19,49,50,51]. Current use of such plants as tinctures in Slovenia has not been recorded in the literature, but this may be due to the lack of research on the topic rather than an actual absence of present use.

The tincture provided the highest alkaloid yield during the present experiments; the results are listed in Table 5. As previously discussed in the water extractions section, this is a large amount of anticholinergic alkaloids with the potential to be quite dangerous. However, it is worth noting that one would not consume the same quantity of a tincture as a water infusion. This would range from a few drops to a small “shot” of the infused alcohol depending on the tradition and thus have a much lower capacity for harm than the water extraction when taken under “normal” use conditions. Whether the tincture were to be taken orally or applied topically would also be an important consideration, and one that we will address in our remarks and limitations section. It is also worth noting that the ethanol used for this experiment was stronger than that which would traditionally be used in making tinctures.

### 3.6. “Smoking” (Smoke Extraction)

Smoking of plants to make use of their anticholinergic tropane alkaloids has a long history and is arguably the manner that has seen the most use over the last century since these plants (primarily *Atropa belladonna* and *Datura stramonium*) were commonly used in commercial smoking blends for asthma; these mixtures were also often abused recreationally, ultimately leading to their discontinuation [52,53,54].

We were forced to switch from the root powder we had used for all other experiments in the present research to leaf powder as the root proved rather difficult to burn. As such, the results from this preparation method would be less comparable to the others. This, however, proved not to be a worry as our extractions and analyses from smoke yielded absolutely no alkaloids, despite going as high as 2.5 g of dried material in our tests; the results are listed in Table 6. Smoking of these plants is known both in the literature on medicinal plants as well as toxicology cases surrounding their abuse, so smoking surely must introduce at least some alkaloids into the human body. This left us with three possible theories: (1) our method was not effective for extracting from smoke. (2) the alkaloids were present in the smoke, but in quantities below the threshold of detection. That being said, when it is known that smoking such plants has intoxicating effects, we would expect to see a much higher level of the alkaloids, especially after burning through 2.5 g of the dried leaves (likely more than an individual would smoke on their own), though how effectively the lungs process these alkaloids and what amount they would need in order to produce effects is unknown. (3) the alkaloids are transformed when burned and thus we would not find them as scopolamine or atropine. Perhaps burning breaks them down to one of their chemical derivatives that still has both medicinal and psychoactive properties. Each of these are, however, simply educated guesses. Further research is needed to prove which (if any) of them are correct.

### 3.7. Remarks and Limitations

It is worth noting that to make the different extraction methods as comparable as possible, we made use of a standardised powdered mix from multiple plant samples. Ethnographic data, however, does not seem to point towards the use of powdered material in most cases. Both for the unguent and for the fortified wine, for example, our previous ethnographic and historical work on this topic has brought up that whole leaves are used. Additionally, we employed *Scopolia carniolica* for all of our experiments due to having an excess of plant material from another study and wishing to keep this degree of standardisation between the extractions. It is worth noting that *Atropa belladonna*, *Datura stramonium*, *Hyoscyamus niger* and *albus*, and *Mandragora* spp. have all been used throughout the years seemingly with much greater frequency than *S. carniolica* for the sorts of products that we have here attempted to recreate. That being said, all contain hyoscyamine/atropine and scopolamine and have been roughly interchangeable in their use.

As such, the current research is not a perfect recreation of traditional methods by any means, but an attempt to merge scientific rigour in experimentation with ethnographic data to determine the effectiveness of these various methods.

It is also worth noting that there are other factors to take into account rather than just the alkaloid content of an extraction. The manner of using the product is of great importance as well. We do not know how effectively our bodies absorb these alkaloids through different organs and thus cannot directly compare beverages and topically applied products. Even the tincture then, which would traditionally be consumed or applied topically depending on the intended use, would actually offer a different amount of alkaloids to our bodies depending on its use despite having the same amount of alkaloids that it was able to extract from the plant material. Due to the potential danger of these alkaloids, further research studying the comparative uptake of these alkaloids in human subjects would need extensive ethical and scientific efforts to ensure the safe acquisition of this knowledge.

## 4. Conclusions

Our results indicated that a tincture (3-day extraction in 70% ethanol) was the most efficient means of extracting these alkaloids. It extracted approximately 70% of the alkaloids present in the plant material. Water was, on the other hand, also a very effective solvent with up to 50% extraction efficacy. This, however, led to a dangerously high amount of alkaloids, which likely explains why teas have been almost exclusively linked to intentional intoxications and toxicology reports rather than more traditional forms of use. Our attempts at recreating unguents did not yield any alkaloids. Our fortified wine extractions generally yielded higher alkaloid concentrations than those done in water and furthermore the social settings in which this beverage would have been traditionally consumed would have made dosing difficult and may have been potentially dangerous given the tendency to overindulge when inebriated. Our attempts to quantify alkaloids in smoke yielded no measurable results, suggesting issues with our method, low yields, or transformation of the alkaloids during burning.

On the whole, our results confirm ethnographic and historical reports of the use of these plants for most of the methods attempted and prove their ability to extract anticholinergic tropane alkaloids from plant material. Future studies focusing on these extraction methods would be of scientific value, especially focusing on those extractions that our research suggests may not actually capture any of the alkaloid content of these plants. Such research should draw on both ethnobotanical and chemical literature to create viable experimental conditions for examining the efficacy of folk preparations for anticholinergic plants of the Solanaceae family.

## Figures and Tables

**Table 1:** The content of atropine and scopolamine as determined by control extractions.

	Atropine Content (µg/g)	Scopolamine Content (µg/g)
Control root mix	2170	330
Control leaf mix	5984	5047

**Table 2:** “Tea” extraction results (% of alkaloid extracted from herbal drug).

			Atropine Extraction Efficacy (%)	Scopolamine Extraction Efficacy (%)
“Tea”	Hot Water	10 min	26.5	37.8
		20 min	40.8	48.4
		30 min	37.5	49.8
	Cold water	10 min	29.1	25.7
		20 min	37.5	47.7
		30 min	35.0	15.5

**Table 3:** “Unguent” extraction results (% of alkaloid extracted from herbal drug).

		Atropine Extraction Efficacy (%)	Scopolamine Extraction Efficacy (%)
“Unguent”	10 min	/	/
	20 min	/	/
	30 min	/	15.3
	Re-extraction	37.7	38.6
	Basified 30 min	/	/

**Table 4:** “Fortified wine” extraction results (µg/mL in the HPLC-ready sample).

		Atropine Extraction Efficacy (%)	Scopolamine Extraction Efficacy (%)
“Fortified wine”	10 min	55.9	38.4
	20 min	59.3	42.3
	30 min	62.8	65.0

**Table 5:** “Tincture” extraction results (µg/mL in the HPLC-ready sample).

	Atropine Extraction Efficacy (%)	Scopolamine Extraction Efficacy (%)
“Tincture”	67.2	71.4

**Table 6:** “Smoking” extraction results (µg/mL in the HPLC-ready sample).

	Atropine Extraction Efficacy (%)	Scopolamine Extraction Efficacy (%)
“Cigarette” Root 0.25 g	/	/
“Cigarette” Root 1 g	/	/
“Cigarette” Leaf 0.25 g	/	/
10 × Leaf 0.25 g “Cigarettes”	/	/
“Pipe” Leaf 2.5 g	/	/

## Data Availability

Data generated in this research is presented here. Additional details can be obtained from the authors by request.
